# The Remediation of Dysprosium-Containing Effluents Using Cyanobacteria *Spirulina platensis* and Yeast *Saccharomyces cerevisiae*

**DOI:** 10.3390/microorganisms11082009

**Published:** 2023-08-04

**Authors:** Inga Zinicovscaia, Nikita Yushin, Dmitrii Grozdov, Alexandra Peshkova, Konstantin Vergel, Elena Rodlovskaya

**Affiliations:** 1 Department of Nuclear Physics, Joint Institute for Nuclear Research, Joliot-Curie Str., 6, 1419890 Dubna, Russia; ynik_62@mail.ru (N.Y.); dsgrozdov@rambler.ru (D.G.); peshkova.alexandra92@gmail.com (A.P.); verkn@mail.ru (K.V.); 2 Department of Nuclear Physics, Horia Hulubei National Institute for R&D in Physics and Nuclear Engineering, 30 Reactorului Str., MG-6, 077125 Magurele, Romania; 3 N. Nesmeyanov Institute of Organoelement Compounds of Russian Academy of Sciences, Vavilova Str., 28, 119991 Moscow, Russia; ro745@mail.ru

**Keywords:** *Saccharomyces cerevisiae*, *Spirulina platensis*, bioremediation, pollution

## Abstract

Dysprosium is one of the most critical rare earth elements for industry and technology. A comparative study was carried out to assess the biosorption capacity of cyanobacteria *Spirulina platensis* and yeast *Saccharomyces cerevisiae* toward dysprosium ions. The effect of experimental parameters such as pH, dysprosium concentration, time of contact, and temperature on the biosorption capacity was evaluated. Biomass before and after dysprosium biosorption was analyzed using neutron activation analysis and Fourier-transform infrared spectroscopy. For both biosorbents, the process was quick and pH-dependent. The maximum removal of dysprosium using *Spirulina platensis* (50%) and *Saccharomyces cerevisiae* (68%) was attained at pH 3.0 during a one-hour experiment. The adsorption data for both biosorbents fitted well with the Langmuir isotherm model, whereas the kinetics of the process followed the pseudo-second-order and Elovich models. The maximum biosorption capacity of *Spirulina platensis* was 3.24 mg/g, and that of *Saccharomyces cerevisiae* was 5.84 mg/g. The thermodynamic parameters showed that dysprosium biosorption was a spontaneous process, exothermic for *Saccharomyces cerevisiae* and endothermic for *Spirulina platensis.* Biological sorbents can be considered an eco-friendly alternative to traditional technologies applied for dysprosium ion recovery from wastewater.

## 1. Introduction

Dysprosium is among the five most important rare earth elements for industry. Its compounds are widely applied in high-tech industries, especially for the production of permanent magnets, wind turbines, electric vehicles, multilayer ceramic capacitors, computer hard disks, windmills, and catalysts [1,2,3,4]. Dysprosium is also used to make nuclear reactor control rods [5]. It is predicted that the demand for dysprosium will increase by 2600% over the next 20 years [1,6]. The price of rare metals on the global market has recently grown significantly as a result of restrictions imposed by China, the world’s largest producer of rare earth elements [4,6]. As a result, the need for their recovery from wastewater and discarded high-tech products has become very relevant.

It is important to note that mining, the processing of rare earth elements, and waste disposal may result in the development of various diseases among local inhabitants and production employees, as well as pollution of the water, air, and soil and the devastation of natural ecosystems [7]. Several studies reported the toxic effect of dysprosium on aquatic organisms [8,9,10]. Therefore, efficient rare earth element recovery is crucial for achieving both economic and sustainable development goals [4].

Hydrometallurgical methods [11,12], solvent extraction [8,13], precipitation, adsorption [5,14,15], and ion exchange techniques [3] have been demonstrated to be particularly effective for the recovery of rare elements. However, the high cost, energy requirement, and generation of toxic sludge could be considered important drawbacks of many of the mentioned techniques [16]. Due to its high efficiency, cost-effectiveness, biosorbent reuse, quick operating time, and lack of hazardous chemical use, biosorption has recently emerged as an economical and environmentally friendly alternative to existing traditional approaches [4,17]. Biosorption involves the application of different types of biological objects for heavy metal and rare earth element recovery [18,19,20].

There is little published information devoted to dysprosium ion biosorption. Devi and Mishra [3] applied raw and modified bark powder of *Mangifera indica* for dysprosium biosorption. The maximum adsorption capacity of 55.04 mg/g was obtained for bark powder treated with NaOH. Two living marine macroalgae, *Gracilaria* sp. and *Ulva lactuca,* used for dysprosium recovery were able to remove, respectively, 89% and 84% of metal ions from the batch solutions [21]. Studying the biosorption of dysprosium on yeast *Saccharomyces cerevisiae* and two aluminum-tolerant yeast strains, Alt-OF2 and Alt-OF5, the authors showed that the latter was able to uptake two times more ions [4]. Lewis and Guéguen [22] applied *Euglena gracilis* for dysprosium removal from solutions containing metal ions in concentrations up to 100 µg/L. Biomass was able to remove 93% of dysprosium ions at pH 3.0.

The use of yeast and cyanobacterial biomass is actively explored for heavy metals, including rare earth elements’ removal from batch systems and real wastewater [23,24,25,26,27,28]. Thus, wild and rim20∆ *Saccharomyces cerevisiae* strains showed high biosorption capacities toward lanthanum ions, 40 and 70 mg/g, respectively [29]. Phosphorylated *Saccharomyces cerevisiae* was applied for the selective adsorption of rare earth elements from a mixture of metals. The biomass removed 50–70% of rare earth elements’ ions from the solution, showing a high preference for Nd and Yb ions [30]. Twelve terrestrial and aquatic cyanobacterial strains were screened for their ability to adsorb Ce, Nd, Tb, and La. Cyanobacterium *Nostoc* sp. 20.02 with an adsorption capacity in the range of 84.2–91.5 mg/g was selected as a promising candidate for rare earth elements’ recovery [19]. Two strains of *Spirulina platensis,* LEB-18 and LEB-52, were employed for Nd removal from aqueous solutions. Both strains showed high adsorption capacities for the target element: 72.5 mg/g for LEB-18 and 48.2 mg/g for LEB-52 [31]. The maximum adsorption of Ce by wild and commercial strains of *Spirulina platensis* amounted to 18.1 and 38.2 mg/g, respectively [32].

The current work aimed to examine the biosorption capacity of cyanobacteria *Spirulina platensis* and yeast *Saccharomyces cerevisiae* toward dysprosium ions. Both biosorbents are characterized by high removal efficiency, ease of cultivation on a large scale, high biomass production, and safety [33]. *Saccharomyces cerevisiae* biomass is obtained in vast quantities as a production waste of the brewing and food industries, while *Spirulina platensis* can be a waste product of the manufacture of bioactive chemicals. To meet the study objectives, the effect of pH, dysprosium concentration, contact time, and temperature on the removal efficiency of biological sorbents was studied. The mechanisms of biosorption were described using kinetic and equilibrium models, while thermodynamic calculations were performed in order to understand the nature of the process.

## 2. Materials and Methods

### 2.1. Chemicals

The chemicals used in the present study Dy(NO_3_)_2_·6H_2_O, HNO_3_, and NaOH were of analytical grade (Sigma-Aldrich, Darmstadt, Germany).

### 2.2. Biosorbents

The yeast *Saccharomyces cerevisiae* (yeast) biomass used as biosorbent was obtained from the residues generated by a brewing company (Chisinau, Republic of Moldova). The cyanobacteria *Spirulina platensis* (spirulina) biomass was purchased from the “Biosolar MSU” company (Moscow, Russia). Before use, both biosorbents were dried at 105 °C for 24 h and homogenized at 600 rpm for 10 min.

### 2.3. Biosorption Experiments

A series of batch experiments were carried out in order to investigate the impact of pH, contact time, temperature, and the dysprosium initial concentration on the biosorption capacity. The pH of the experimental solutions ranged from 2.0 to 6.0, the time of contact ranged from 5 to 120 min, the dysprosium concentration ranged from 10 to 100 mg/L, and the temperature ranged from 20 to 50 °C. The experiments were performed by mixing 0.5 g of biosorbent with 50 mL of experimental solution at a constant stirring speed of 200 rpm. The kinetic experiments were carried out by varying the contact time and keeping the dysprosium concentration (10 mg/L), temperature (20 °C), and pH-value (3.0) constant. The effect of the pH on biosorption was studied by changing it to the desired values using nitric acid or sodium hydroxide. Adsorption equilibrium and thermodynamic experiments were carried out in accordance with the abovementioned procedure, varying the parameter of interest. At the end of the experiments, the biomass separated from the solution was dried at 105 °C, weighed using an analytical balance, and packed in polyethylene bags for elemental analysis. Experiments were performed in triplicate. 

### 2.4. Biosorbent Analysis

To assess the dysprosium biosorption as well as the changes in the content of magnesium, chlorine, calcium, and manganese in biomass samples, neutron activation analysis was performed using a pulsed fast reactor IBR-2 (Frank Laboratory of Neutron Physics, Joint Institute for Nuclear Research, Dubna, Russia). The samples were irradiated for 3 min at a neutron flux of 1.2 × 10^12^ n·cm^−2^ s^–1^ and measured immediately after irradiation for 15 min. The quality control of the analytical measurements was carried out using certified reference materials (National Institute of Standards and Technology, Gaithersburg, MD, USA): NIST SRM 2709—San Joaquin Soil, NIST SRM 1547—Peach Leaves, and NIST SRM 1575a—Trace Elements in Pine Needles.

The efficiency of dysprosium removal was calculated using Equation (1) as follows:

(1) $$R=\frac{C_{i}-C_{f}}{C_{i}}\times 100$$

where C_i_ and C_f_ are the initial and final concentrations of dysprosium in solution (mg/L).

The involvement of functional groups in dysprosium ion binding was confirmed via Fourier-transform infrared spectroscopy (FTIR). The spectra were recorded using a Nicolet 6700 spectrometer (Thermo Scientific, Waltham, MA, USA).

## 3. Results and Discussion

### 3.1. The Effect of Experimental Parameters on Dysprosium Ion Removal

**Figure 1:**
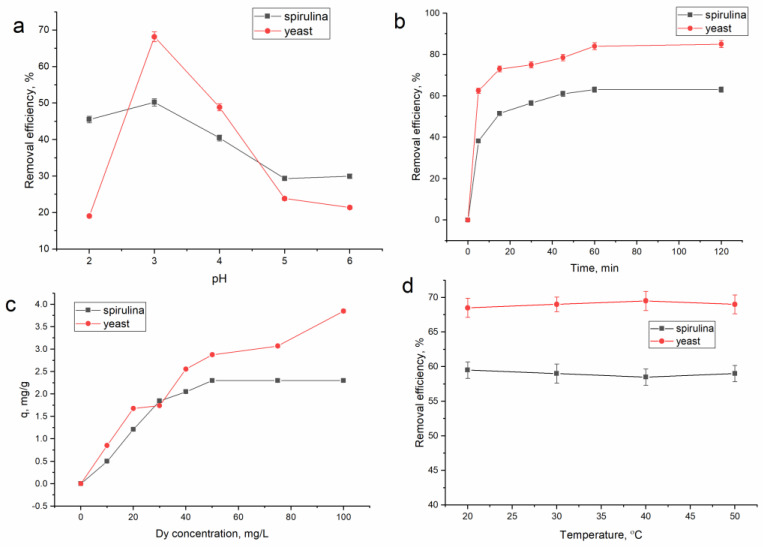
Influence of experimental parameters on dysprosium ion removal: (**a**) pH, (**b**) time, (**c**) dysprosium concentration, and (**d**) temperature.

The pH level has a considerable impact on the biosorption of metal ions by altering the characteristics of both the adsorbent and the adsorbate [34]. The effect of pH on dysprosium ion removal was examined in the pH range from 2.0 to 6.0 (Figure 1a). Only 19% of the dysprosium ions were removed from the solution at pH 2.0 when using the yeast biomass, compared with a removal efficiency of 45% obtained for spirulina biomass. At pH 2.0, protons compete with dysprosium cations for binding sites, making metals’ sorption difficult [35]. Maximum dysprosium adsorption occurred at pH 3.0 for both biosorbents: 0.50 mg/g for spirulina (50% removal) and 0.68 mg/g (68% removal) for yeast. High biosorption at a specified pH value can be associated with the increase in the number of adsorption sites that can be used to adsorb dysprosium ions, thereby facilitating the adsorption process. With an increase in pH, the surface of biosorbents becomes more negatively charged, resulting in an increase in its affinity to metal ions in cationic form. A further increase in pH was associated with a decrease in biosorbents’ removal capacities, up to 30% for spirulina at pH 6.0 and 21% for yeast at the same pH value.

Dy^3+^ is the major form of dysprosium at pH values between 1.0 and 4.0, while at higher pH values, other species such as Dy(OH)^2+^, Dy(OH)_2_^+^, and Dy(OH)_3_ are formed [5,36]. The decrease in dysprosium removal at higher pH values can be explained by the formation of Dy(OH)_3_, which inhibits the biosorption process [37]. Thus, further experiments were performed at pH 4.0.

The highest percentages of dysprosium adsorption on raw bark powder, HDTMA-Br-treated bark powder, and NaOH-treated bark powder of 48, 66, and 71, respectively, were achieved at initial pH values of 2.0 and 3.0 [3]. At pH 4.0, two types of activated carbons (AC-CA and AC-PA) derived from used coffee grounds were able to remove 94% and 100%, respectively, of dysprosium ions [1]. The highest dysprosium adsorption onto carbonized mandarin orange peels (100%) and ginkgo leaves (86%) was attained at pH levels of 3.0 and 5.0, respectively [38]. Dysprosium adsorption onto two Al-tolerant yeast strains, Alt-OF2 and Alt-OF5, was most effective at pH 6.0 [4]. A hybrid donor functionalized alumina-silica-based nanomaterial exhibited maximum dysprosium adsorption of 125.44 mg/g at pH 4.0 [5].

The removal of metal ions from aqueous solutions is strongly influenced by two factors: time and metal concentration in the solution. Studying the effect of contact time on the efficiency of dysprosium removal (Figure 1b), it was found that fast metal ions’ biosorption occurred within the first five minutes of the reaction when 38% of dysprosium ions were removed with spirulina and 62.5% with the yeast biomass. The highest dysprosium adsorption was accomplished after 60 min, reaching 66% for spirulina and 85% for yeast. The fast dysprosium ion biosorption at the beginning of the reaction is explained by the availability of a large number of binding sites on the biosorbent surface [39,40], and its lowering at the next stage is associated with the saturation of metal binding sites and equilibrium attainment [37]. It is believed that fast metal ion removal in the first stage is mainly associated with surface adsorption, while in the second stage (the slow one), gradual adsorption is dominant [41]. At the same time, it is important to mention that the quick achievement of equilibrium may indicate that biosorption is a typical physicochemical interaction between biomass and metal ions [42].

The biosorption capacity of the yeast biomass (0.85 mg/g) was higher than that of spirulina (0.66 mg/g) and can be explained by the differences between the functional groups on the biomass surface. In the case of *Spirulina platensis* and *Saccharomyces cerevisiae* applications for silver ions’ removal from aqueous solutions, the same pattern was observed [43,44]. *Spirulina platensis* adsorbed 2.54 mg/g of silver ions, while *Saccharomyces cerevisiae* adsorbed 3.48 mg/g of silver ions. 

Figure 1c shows the impact of the initial dysprosium concentration on its biosorption. The yeast biosorption capacity continuously increased from 0.85 to 3.85 mg/g, while the dysprosium concentration increased from 10 to 100 mg/L. An increase in the driving force in mass transfer operations between the aqueous and solid phases with the increase in the initial concentration can be used to explain the high adsorption capacity of yeast at growing dysprosium concentrations [45]. For the yeast biomass, no saturation occurred at the studied range of dysprosium concentrations. The highest adsorption capacity of spirulina was reached at a dysprosium concentration of 50 mg/L, and it remained constant even as the concentration of metal in the solution increased. This effect can be explained by the saturation of adsorption sites [40]. However, it should be mentioned that in the case of spirulina biomass use for other rare earth elements’ biosorption at the same range of metal concentrations, a continuous increase in the biomass sorption capacity was found [23,46].

Figure 1d illustrates the temperature effect on the removal of dysprosium ions. The removal of dysprosium using spirulina and yeast biomass was almost not affected by the change in temperature. Thus, the removal capacity of spirulina at the temperature range of 20–50 °C was 59%, and that of the yeast biomass was 69%. Previously, it was shown that temperature almost did not affect the rate of Er removal using spirulina biomass [47], which was on the level of 61–68% at a temperature range of 20–50 °C. The temperature also did not play an essential role in dysprosium removal with the activated carbon obtained from spent coffee waste [1], and it was at the level of 80%.

### 3.2. Experimental Data Evaluation

**Figure 2:**
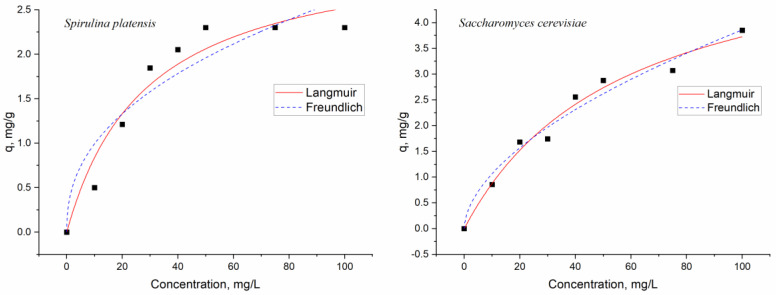
Description of the equilibrium experimental data applying Langmuir and Freundlich models (black squares are experimental data).

Adsorption isotherms are useful for understanding the adsorption mechanism. The equilibrium of dysprosium biosorption onto spirulina and yeast biomass was examined by applying nonlinear forms of Langmuir and Freundlich isotherm models (Figure 2).

The fundamental assumption of the Langmuir model is that metal ion uptake occurs on a homogeneous surface via monolayer adsorption without any interaction between adsorbed metal ions, i.e., all binding sites have the same affinity for adsorbates, and adsorption at one site does not affect adsorption at an adjacent site [48]. The Freundlich isotherm model, empirical in nature, is applied to describe adsorption on heterogeneous surfaces. The model assumes that strong binding sites are occupied first, and the level of site occupation increases with the decline in binding strength [48,49]. Equilibrium data were defined using Equations (2) and (3).

(2) $$q_{m}=\frac{q_{m}bC_{e}}{1+bC_{e}}$$

(3) $$q_{m}=K_{F}{Ce}^{\frac{1}{n}}$$

where C_e_ is the dysprosium concentration at equilibrium (mg/L); q_m_ is the maximum content of adsorbed dysprosium (mg/g); b is s the Langmuir constant related to the adsorption energy (L/mg); K_F_ and n are Freundlich model constants related to adsorption capacity and adsorption intensity, respectively.

The separation factor R_L_ was calculated using Equation (3).

(4) $$R_{L}=\frac{1}{1+bC_{i}}$$

An R_L_ value of less than one unit indicates that adsorption is favorable, and R_L_ values higher than one unit mean that adsorption is unfavorable.

The parameters of the applied models are summarized in Table 1.

An examination of the correlation coefficients suggested that the Langmuir model was more suitable to present the experimental data for both biosorbents. The Langmuir model allows for the estimation of the maximum theoretical metal uptake capacity, which could not be reached in the experiments. Thus, the maximum biosorption capacity for yeast biomass was 5.84 mg/g, and it was 1.8 times higher than for spirulina biomass. According to *b* values, a high affinity of the sorbent to sorbate was noted. The separation factor (R_L_) values of 0.76 for spirulina and 0.85 for yeast also supported this fact. The applicability of the Langmuir model suggests homogeneous biosorption: Once an adsorbate molecule occupies a site, no more adsorption may occur there. When compared to the Langmuir isotherm model, the Freundlich isotherm model’s correlation coefficients were found to be lower. The favorable nature of the dysprosium biosorption on the heterogeneous surfaces of the analyzed biosorbents was demonstrated with the n values from Freundlich models, which were between 1.0 and 10 [50].

The maximum adsorption capacities obtained in the present study were compared with those of other sorbents, mainly produced synthetically (Table 2). It can be seen that the adsorption capacity of the synthesized adsorbents was higher, but the low cost of the biological sorbents justifies their use for wastewater treatment. It is also important to mention that adsorption capacity depends on experimental conditions, which were different in the presented studies.

**Figure 3:**
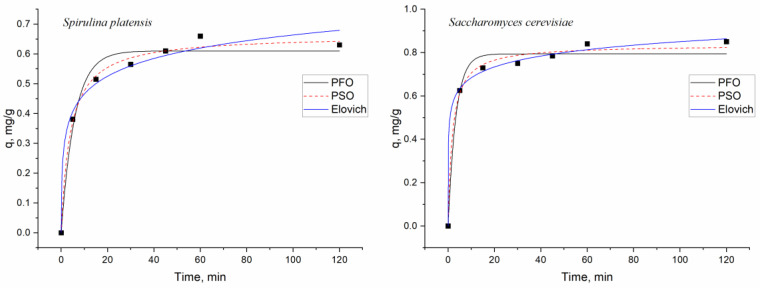
Description of the kinetics of the process by applying pseudo-first order, pseudo-second order, and Elovich models (black squares are experimental data).

Adsorption kinetics provides information on the chemical pathways and mechanisms involved in the sorbate adsorption on the sorbent [50]. The kinetics of biosorption was explained using nonlinear forms of pseudo-first-order, pseudo-second-order, and Elovich models (Figure 3), expressed using Equations (5)–(7).

The pseudo-first-order (PFO) model demonstrates that the adsorption capacity is determined by the rate at which ions bind to the adsorbent [48]. It was developed for sorption in liquid–solid systems and is based on solid capacity [42].

(5) $$q_{t}=q_{e}(1-e^{-k_{1}t})$$

The pseudo-second-order (PSO) model is applicable for the description of chemical adsorption, which involves valence forces through the sharing or exchange of electrons between the metal ions and biosorbent:

(6) $$q=\frac{q_{e}^{2}k_{2}t}{1+q_{e}k_{2}t}$$

The Elovich model (EM) is used to describe chemical adsorption and is applicable to heterogeneous surfaces. The model assumes that the rate of biosorption decreases exponentially with an increase in the amount of adsorbate [52,53].

(7) $$q_{t}=\frac{1}{\beta }ln(1+\alpha \beta t)$$

where q_t_ is the amount of dysprosium adsorbed (mg/g) at time t (mg/g); k_1_ (1/min is the rate constant of first-order adsorption; k_2_ (g/mg·min) is the rate constant of second-order adsorption; and α (g/mg∙min) and β (g/mg) are Elovich model constants.

A comparison of the coefficients of correlation values showed that pseudo-second-order and Elovich models could be applied to describe the kinetics of the dysprosium ions’ biosorption onto both analyzed sorbents (Table 3). The computed q_e_ values from the pseudo-second model were in good agreement with the experimental values. Low k_1_ values for the pseudo-first-order model indicate a slow adsorption process, while high k_2_ values for the pseudo-second-order model point to an increase in adsorption rate. The mechanism of metal ions’ biosorption depends on (i) the physical and chemical properties of the biosorbent and (ii) the mass transfer process from the adsorbate onto the adsorbent [42]. The applicability of the pseudo-second-order and Elovich models indicates the chemical adsorption of dysprosium biosorption on both biosorbents, characterized by the exchange of electrons between adsorbent and adsorbate [34]. It should be mentioned that the values of kinetic parameters for the pseudo-first-order model were marginally lower than those obtained for the pseudo-second-order model, indicating that besides chemical interaction, the diffusion of the ions onto the active sites may also influence the kinetics of the process [42].

The thermodynamic parameters provide in-depth information about the energetic changes associated with the adsorption process [3]. The thermodynamic parameters such as the standard free energy (ΔG°), enthalpy change (ΔH°), and entropy change (ΔS°) were computed from Equations (8)–(10) as follows:

(8) $$lnK_{d}=\frac{\Delta S{}^{\circ}}{R}-\frac{\Delta H{}^{\circ}}{RT}$$

(9) ΔG°=ΔH°−TΔS°

The distribution coefficient K_d_ was calculated using Equation (10) as follows:

(10) $$K_{d}=\frac{C_{a}}{C_{e}}$$

where C_a_ is the concentration of dysprosium adsorbed (mg/L R) is the universal gas constant (8.314 J/moK), and T is the absolute temperature (K).

The values of ΔH° and ΔS° were determined by the slope and the intercept from the plot of lnK_d_ against 1/T (Figure S1). The thermodynamic values are given in Table 4.

The negative ΔG° values at all studied temperatures indicate the spontaneous nature of the adsorption of dysprosium on biological sorbents. The negative value of ΔH° shows that the dysprosium biosorption onto yeast biomass was exothermic in nature, while on spirulina biomass—endothermic. At ΔH° values lower than 40 kJ/adsorption is considered a physical process, while at values in the range of 80–200 kJ/mol is a chemical process [47]. The ΔS° values were positive for both biosorbents and show the affinity of the adsorbent for the dysprosium ions, indicating an increase in sorbate concentration in the solid–liquid interface [50].

### 3.3. Mechanisms of the Dysprosium Biosorption

Using neutron activation analysis, it was possible to determine the content of magnesium, calcium, chlorine, and manganese in raw and dysprosium-supplemented biomass. In the spirulina biomass after dysprosium ions’ biosorption, the content of magnesium decreased by 38%, the content of chlorine decreased by 86%, the content of calcium decreased by 28%, and the content of manganese decreased by 17%. In the case of the yeast biomass, the content of magnesium was reduced by 66%, chlorine was reduced by 96%, and manganese was reduced by 38%. The content of calcium in biomass samples remained unchanged. Thus, ion exchange is one of the mechanisms involved in dysprosium removal by biological sorbents. 

**Figure 4:**
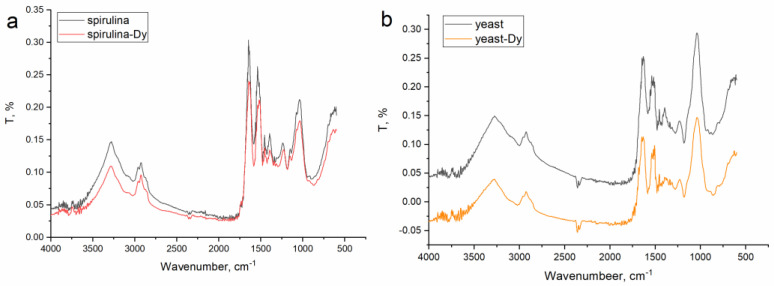
FTIR spectra of control and dysprosium-loaded biomass: (**a**) spirulina and (**b**) yeast.

FTIR analysis was applied to assess the participation of functional groups in dysprosium ion binding (Figure 4). In the control spectrum of the spirulina biomass (Figure 4a), intensive bands at 3260 and 2950 cm^−1^ corresponding to amine (–NH) and OH groups were observed. The peaks at 1214 cm^−1^ and 1730 cm^−1^ are attributed to the stretching vibration of carboxyl (C=O) groups, while the peak at 1525 cm^−1^ corresponds to the stretching vibration of alkyl groups. Adsorption peaks in the region 1650–1200 cm^−1^ could also be attributed to the C–O, –C–C, and –C–OH groups of proteins [28]. The decrease in the bands’ intensity and their slight shift indicate the involvement of C=O, C–O, –C–C, and –C–OH groups in dysprosium capture. 

**Figure 5:**
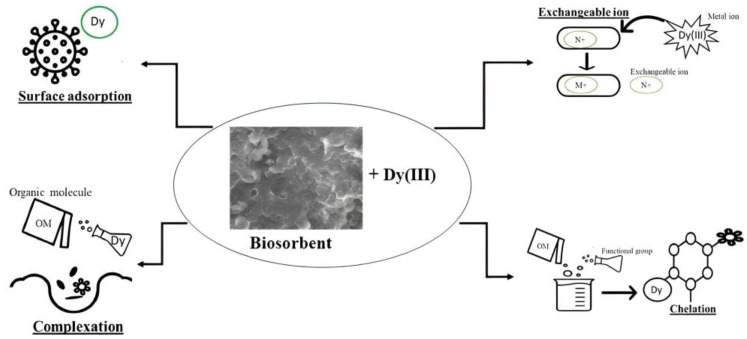
Scheme of biosorbents’ interaction with dysprosium ions.

The analysis of the yeast spectrum (Figure 4b) revealed intensive bands at 3550, 2975, and 1020 cm^−1^ attributed to amine (−NH) and OH groups. Absorption bands in the regions at 1040 and 1520 cm^−1^ correspond to OH groups, while the bands at 1393 and 2950 cm^−1^ represent the stretching vibration of CH_3_ or CH_2_ groups. The peak at 1525 cm^−1^ is related to the vibration of aromatic groups, and the peak at 1625 cm^−1^ is attributed to CH=CH groups. The peak at 1525 cm^−1^ is related to the vibration of aromatic groups, and the absorption band at 1626 cm^−1^ corresponds to CH=CH groups. The peak of the symmetrical stretching vibration of the phosphodiester group [−${PO}_{2}^{-}$] was observed at 1092 cm^−1^. In addition, in the region at 1650–1200 cm^−1^, the stretching vibrations of –C–O, –C–C, and –C–OH groups were observed [28]. In the dysprosium-loaded spectrum, no significant changes were observed; however, a reduction in the bands’ intensity can be associated with the dysprosium ion binding, which results in the occurrence of bond stretching to a lesser degree [28]. The OH, C=O, and C–O groups were involved in the biosorption of dysprosium onto raw bark powder [3]. Dysprosium biosorption can also occur via the electrostatic interaction of metal ions with P–OH groups [54]. The possible mechanisms of dysprosium ions’ interactions with biosorbents are presented in Figure 5.

## 4. Conclusions

The results obtained in the present study demonstrate that *Spirulina platensis* and *Saccharomyces cerevisiae* can be used as economically and environmentally friendly biosorbents for dysprosium removal from wastewater. The dysprosium biosorption was dependent on the contact time, pH, and initial dysprosium concentrations, while temperature did not affect the rate of metal ion removal. The maximum dysprosium removal of 50% with *Spirulina platensis* and 68% with *Saccharomyces cerevisiae* was achieved at pH 3.0 during a 1 h experiment. The kinetics of biosorption was better described using pseudo-second-order and Elovich models. Equilibrium data fitted well to the Langmuir model, with a maximum adsorption capacity of 3.24 mg/g for *Spirulina platensis* and 5.84 mg/g for *Saccharomyces cerevisiae.* The thermodynamic study showed that the process of dysprosium biosorption was spontaneous and exothermic for *Saccharomyces cerevisiae* and endothermic for *Spirulina platensis*. Ion exchange and metal ion binding to functional groups are the main mechanisms of dysprosium adsorption onto yeast and spirulina biomass. *Saccharomyces cerevisiae* can be considered a preferable biosorbent for dysprosium recovery due to its higher adsorption capacity and large availability.

## Figures and Tables

**Table 1:** Parameters of Langmuir and Freundlich models applied to describe dysprosium biosorption using spirulina and yeast biomass.

Isotherms						
Biosorbent	Langmuir			Freundlich		
	q_m_	b	R^2^	K_F_	n	R^2^
*Spirulina platensis*	3.24 ± 0.42	0.03 ± 0.01	0.95	0.37 ± 0.16	2.36 ± 0.61	0.89
*Saccharomyces cerevisiae*	5.84 ± 0.68	0.017 ± 0.004	0.98	0.29 ± 0.07	1.78 ± 0.18	0.97

**Table 2:** Comparison of maximum sorption capacity of different sorbents toward dysprosium ions.

Sorbent	pH	q, mg/g	Reference
*Spirulina platensis*	3.0	3.24	Present work
*Saccharomyces cerevisiae*	3.0	5.84	Present work
AC-CA	4.0	28.11	[1]
Imprinted mesoporous silica	2.0	27.24	[6]
AC-PA	4.0	29.05	[1]
*Mangifera indica*	3.0	92	[3]
Dowex 50WX8	3.0	50	[51]
*Euglena gracilis*	3.0	0.027	[22]
Hybrid donor functionalized alumina-silica-based nanomaterial	4.0	125.44	[5]

**Table 3:** Parameters of kinetic models applied to describe dysprosium biosorption by spirulina and yeast biomass.

Kinetics									
Sorbent	Pseudo-First Order			Pseudo-Second Order			Elovich		
	q_e_	k_1_	R^2^	q_e_	k_2_	R^2^	α	β	R^2^
*Spirulina platensis*	0.61 ± 0.01	0.17 ± 0.03	0.97	0.66 ± 0.01	0.38 ± 0.06	0.99	1.91 ± 1.64	11.6 ± 1.7	0.98
*Saccharomyces cerevisiae*	0.79 ± 0.02	0.29 ± 0.05	0.98	0.83 ± 0.01	0.64 ± 0.13	0.99	91.50 ± 8.82	13.8 ± 1.3	0.99

**Table 4:** Thermodynamic parameters for dysprosium biosorption at different temperatures.

Sorbent	Temperature, K	ΔG°, kJ/mol	ΔH°, kJ/mol	ΔS°, J/mol·K
*Spirulina platensis*	293	−9.8	89	34
	303	−10.2		
	313	−10.6		
	323	−11.0		
*Saccharomyces cerevisiae*	293	−10.3	−91	35
	303	−10.6		
	313	−11		
	323	−11.3		

## Data Availability

The data presented in this study are available on request from the corresponding author.

## Supplementary Materials

The following supporting information can be downloaded at: https://www.mdpi.com/article/10.3390/microorganisms11082009/s1, Figure S1: lnK_d_ versus 1/T.
