# Supplementary figures and images for: The Remediation of Dysprosium-Containing Effluents Using Cyanobacteria Spirulina platensis and Yeast Saccharomyces cerevisiae
Source: Microorganisms. 2023 Aug 4;11(8):2009. doi: 10.3390/microorganisms11082009 (PMC10458459; doi:10.3390/microorganisms11082009)

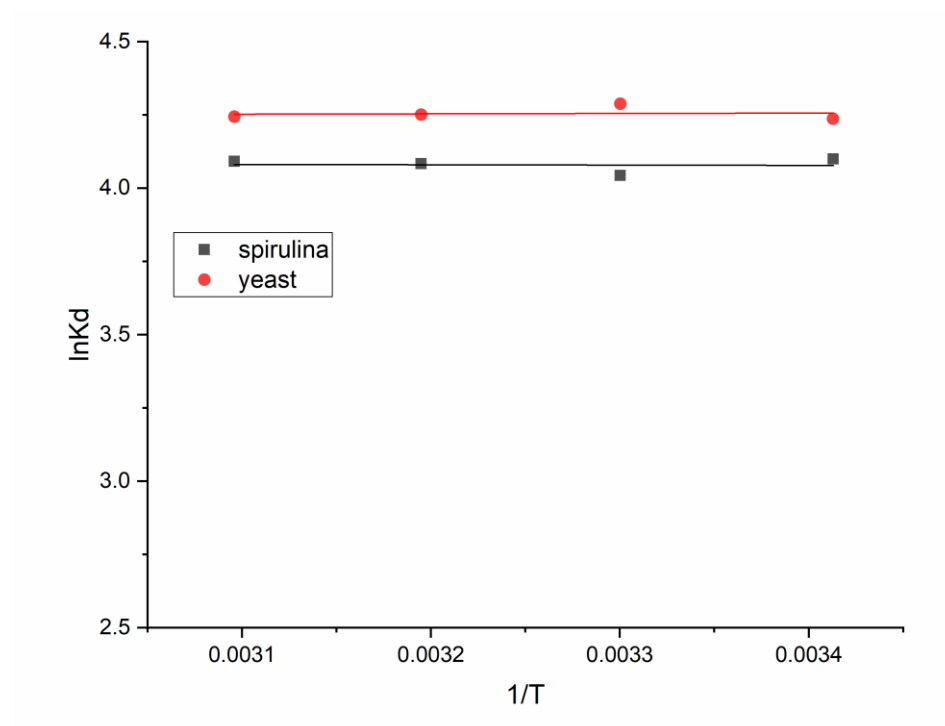

**Figure S1.**  $\ln K_d$  versus  $1/T$

Supplement: Supplementary file 1 [file microorganisms-11-02009-s001.zip › microorganisms-2534396-supplementary.pdf]
